# Effect of Biofilm Formation by *Lactobacillus plantarum* on the Malolactic Fermentation in Model Wine

**DOI:** 10.3390/foods9060797

**Published:** 2020-06-17

**Authors:** Gianfranco Pannella, Silvia Jane Lombardi, Francesca Coppola, Franca Vergalito, Massimo Iorizzo, Mariantonietta Succi, Patrizio Tremonte, Caterina Iannini, Elena Sorrentino, Raffaele Coppola

**Affiliations:** 1 Department of Agricultural, Environmental and Food Sciences (DiAAA), University of Molise, via De Sanctis snc, 86100 Campobasso, Italy; gianfranco.pannella@unimol.it (G.P.); silvia.lombardi@unimol.it (S.J.L.); franca.vergalito@unimol.it (F.V.); succi@unimol.it (M.S.); tremonte@unimol.it (P.T.); iannini@unimol.it (C.I.); sorrentino@unimol.it (E.S.); coppola@unimol.it (R.C.); 2 Department of Agricultural Sciences, Grape and Wine Science Division, University of Naples “Federico II”, Viale Italia, 83100 Avellino, Italy; fracop93@libero.it

**Keywords:** acid stress, ethanol stress, biological decarboxylation, wood attached cells

## Abstract

Biofilm life-style of *Lactobacillus plantarum* (*L. plantarum*) strains was evaluated in vitro as a new and suitable biotechnological strategy to assure L-malic acid conversion in wine stress conditions. Sixty-eight *L. plantarum* strains isolated from diverse sources were assessed for their ability to form biofilm in acid (pH 3.5 or 3.2) or in ethanol (12% or 14%) stress conditions. The effect of incubation times (24 and 72 h) on the biofilm formation was evaluated. The study highlighted that, regardless of isolation source and stress conditions, the ability to form biofilm was strain-dependent. Specifically, two clusters, formed by high and low biofilm producer strains, were identified. Among high producer strains, *L. plantarum* Lpls22 was chosen as the highest producer strain and cultivated in planktonic form or in biofilm using oak supports. Model wines at 12% of ethanol and pH 3.5 or 3.2 were used to assess planktonic and biofilm cells survival and to evaluate the effect of biofilm on L-malic acid conversion. For cells in planktonic form, a strong survival decay was detected. In contrast, cells in biofilm life-style showed high resistance, assuring a prompt and complete L-malic acid conversion.

## 1. Introduction

Malolactic fermentation (MLF) consists of the decarboxylation of tricarboxylic L-malic acid to dicarboxylic L-lactic acid and CO_2_. This bioconversion, known as secondary fermentation, is highly desirable in red wines or in certain white wines and is usually carried out by native lactic acid bacteria (LAB) or by selected LAB cultures [1,2,3,4,5]. Several species belonging to *Lactobacillus*, *Pediococcus*, *Leuconostoc*, and *Oenococcus* genera are able to convert L-malic acid to L-lactic acid, but generally only selected strains of *Oenococcus oeni* are the most common malolactic starter cultures [6,7]. In the last decade, several studies focused their attention on this topic, and LAB species other than *O. oeni* to be used in winemaking and in the MLF process were studied [8,9,10,11,12]. In particular, strains belonging to the *Lactobacillus genus* could also exert an important role in extreme environments owing to their enzyme pathway [13,14,15,16,17,18,19,20]. In fact, *L. plantarum* strains, having genes encoding for enzymes such as citrate lyase, phenolic acid decarboxylase, esterase, and β-glucosidases, could positively influence the MLF and wine flavour [21,22]. The success of MLF mainly depends on the concentration of the starter culture as well as its ability to survive in the prohibitive conditions of wine. In addition, MLF can be spread out over time, increasing the risk of quality decay due to wine oxidation or microbial spoilage [3]. On the other hand, the failure of MLF is generally attributable to rapid cells loss vitality [23]. For this reason, the capability to survive and degrade L-malic acid in acid and ethanol stress conditions represents the most important feature to be screened in the selection process of a potential malolactic strain. Several approaches, ranging from the individuation of resistant strains to stress preadaptation, have been proposed to enhance the malolactic performances [24,25,26,27,28,29,30]. However, many of the adopted strategies could have several limitations. For instance, some *L. plantarum* strains, declared as resistant, convert L-malic acid to L-lactic acid only up to pH 3.6, becoming ineffective at pH values lower than 3.5 [3]. In addition, other strategies, such as stress preadaptation, appear too time-consuming for winemakers. Interesting approaches may derive from biofilm formation by certain *Lactobacillus* strains. In the last few years, biofilm life-style of *Lactobacillus* spp. has gained increased attention. Cells of *L. plantarum* in biofilm form enhance resistance to acid and ethanol stress [31]. However, most of these studies evaluated the biofilm formation of *L. plantarum* in relation to food spoilage or probiotic traits [32,33,34,35,36]. Moreover, the use of biofilms in biotechnological processes, such as waste water treatment or acetic acid production, has been well elucidated [37]. Moreover, the use of extracellular polymeric substances from *L. plantarum* has been proposed in the food industry for their useful properties. However, to date, little information is available on the influence of biofilm life-style of *L. plantarum* strains on malolactic fermentation. To our knowledge, only one study investigated the survival of surface-associated *Oenococcus oeni* cells on MLF [38]. Thus, in the present work, the effect of *L. plantarum* biofilm on the L-malic acid decarboxylation was evaluated. In particular, *L. plantarum* strains were firstly screened for their ability to form biofilm at low pH and high ethanol levels. Subsequently, the survival of *L. plantarum* cells in biofilm life-style and the effect of high-biofilm producing strains on L-malic acid conversion were evaluated in a model wine medium.

## 2. Materials and Methods

### 2.1. Bacterial Strains and Culture Conditions

Sixty-seven *L. plantarum* strains (DiAAA collection, University of Molise), previously isolated from traditional red wines (37), sourdough (19), and bee bread (11), and already characterized for their malolactic activity [11], were used in the present study. The commercial culture *L. plantarum* V22 (Lallemand Inc., Montreal, QC, Canada) was used as reference strain. The strains, stored at −80 °C [16], were propagated twice in de Man Rogosa Sharpe (MRS, Oxoid, Milan, Italy) broth (pH 6.2) at 28 °C prior to their use.

### 2.2. Biofilm Assay

#### 2.2.1. Effect of Environmental Stress Conditions

The strains were screened in order to assess their ability to form biofilm in acid or ethanol stress conditions. For this purpose, each strain was cultivated in MRS broth at 28 °C until the early stationary growth phase was reached. The cells were recovered by centrifugation at 8500 rpm for 15 min, washed twice with phosphate buffered saline (PBS), and resuspended in MRS broth (pH 6.2). Each strain was used to inoculate (final concentration of about 7 Log CFU/mL, corresponding to an optical density at 620 nm -OD_620_- ≃ 0.02) 96-well polystyrene microtiter plates (Thermo Fisher Scientific, Waltham, MA, Unit State) filled with 200 µL of MRS with 12% (Et12) or 14% (Et14) of ethanol (Sigma Aldrich, Milan, Italy) or with MRS acidified with HCl (Sigma Aldrich, Milan, Italy) until pH 3.5 (pH 3.5) or pH 3.2 (pH 3.2); wells filled with MRS at pH 6.2 were used as control. After 2, 24, and 72 h of incubation at 28 °C, the biofilm formation was detected using the crystal violet (CV) assay, as reported by Merritt et al. [39]. Briefly, the medium was removed with a micropipette and the resulting biofilm was washed three times with 230 µL of phosphate buffered saline (PBS) to remove unattached cells. The biofilm was stained with 200 µL of CV (0.1% v/v) solution for 30 min. The excess of CV was removed and the biofilm was washed three times with 230 µL of PBS. The dye attached to biofilm was dissolved with 200 µL of acetic acid (30% v/v) solution for 30 min, and then 100 µL was transferred in a new microtiter plate and OD_620_ was measured (Multiskan FC, Thermo Fisher Scientific, Waltham, MA, Unit State). OD_620_ values from the CV assay were used to determine the biofilm expression index. For this purpose, OD_620_ raw values were structured as numeric matrix, standardized, and elaborated by multivariate analysis. The method used for the biofilm expression is detailed in Section 2.5 (Statistical Analysis).

The growth of planktonic *L. plantarum* strains was also ascertained after 24 h and 72 h of incubation. Microbial growth was followed over time by measuring the optical density at 620 nm (OD_620_). The maximum specific growth rate (μ*_max_*) was calculated by linear regression of Ln (OD/OD_0_) as a function of the time, where OD_0_ is the optical density at the beginning of the exponential growth phase.

In addition, a ratio value (biofilm_score), intended as the ratio between the median values of CV OD_620_ (median values of OD_620_ from CV assay carried out as reported by Merritt et al. [39]) and OD_620_ (median values of OD_620_ calculated on cells in planktonic form), was calculated at 24 and 72 h of incubation.

### 2.3. Quantification of Culturable Cells in the Biofilm

Culturable cells of *L. plantarum* in the biofilms produced in MRS broth without stress conditions (control) or in presence of acid (pH 3.5, pH 3.2) or ethanol (Et12, Et14) stress were quantified using the vital count plate technique. The quantification was carried out for nine *L. plantarum* strains selected on the basis of the ability to produce high quantity of biofilm. The selected strains in the early stationary growth phase were inoculated (final OD_620_ ≃ 0.02) in six-well polystyrene microtiter plates filled with 1 mL MRS without or with acid (pH 3.5, pH 3.2) or ethanol (Et12, Et14) stress conditions. Microtiter plates were incubated for 24 h and 72 h at 28 °C in static conditions. Afterwards the medium was removed from microtiter wells and the biofilm cells, attached on the bottom of wells, were washed two times with PBS and collected with a scraper for microbial count in MRS plates.

### 2.4. Survival and L-malic Acid Conversion by Planktonic or Biofilm Cells in Model Wine

#### 2.4.1. Cells Obtainment in Planktonic and in Biofilm Form

*L. plantarum* Lpls22 was chosen on the basis of its ability to produce biofilm on polystyrene plates and cultivated in planktonic and in biofilm form. To obtain the planktonic cells, *L. plantarum* Lpls22 culture was inoculated (final concentration of about 7 Log CFU/mL) in MRS broth (pH 6.2) at 28 °C for 24 h. The planktonic cells were recovered by centrifugation at 8500 rpm for 15 min and washed twice with PBS. As for cells in biofilm life-style, sterile wood supports (24 cm^2^/support) were used. In particular, *L. plantarum* Lpls22 culture was obtained in MRS broth as previously reported (final concentration of about 7 Log CFU/mL). Oak supports were then immersed in this medium. After incubation for 24 h at 28 °C, the wood supports, containing the biofilm of the strain, were aseptically removed from the culture broth and washed twice by immersion in sterile PBS in order to remove both the residues and the non-adherent cells. Adherent cells concentration per cm^2^ of the wood support was estimated by direct microscopic determination using the Thoma-Zeiss counting chamber (Merck KGaA, Darmstadt, Germany). For this purpose, the adherent cells in form of biofilm were accurately removed by scarping and resuspended in physiological solution (9 g/L NaCl) [33].

#### 2.4.2. Cell Survival and L-malic Acid Conversion

The ability to survive and to convert L-malic acid to L-lactic acid by *L. plantarum* Lpls22 in planktonic or in biofilm form was evaluated in a model system (model wine, MW) at 12% of ethanol and pH 3.5 or pH 3.2. MW was prepared as described by Bravo-Ferrada et al. [40] and inoculated with cells in planktonic or in biofilm life-style adhered to the oak wood supports. For this purpose, Erlenmeyer culture flasks containing 240 mL of MW at 12% of ethanol (pH 3.5, pH 3.2) were inoculated with 10 wood supports (total exhibition area = 240 cm^2^) containing *L. plantarum* Lpls22 in biofilm form. Another two Erlenmeyer culture flasks containing 240 mL of MW at 12% of ethanol (pH 3.5, pH 3.2) were inoculated with planktonic cells in order to obtain the same final concentrations of cells in biofilm form, estimated as previously described.

As additional tests, *L. plantarum* Lpls22 pre-adapted as reported by Succi et al. [29] was inoculated in MW at 12% of ethanol (pH 3.5, pH 3.2) using the same final concentrations of planktonic or biofilm cells.

At the time of the inoculum (time 0), after 24 and 96 h of incubation, aliquots of samples were taken to enumerate pre-adapted and non-pre-adapted planktonic cells, biofilm cells, and detached cells from biofilm. The L-malic acid conversion was evaluated at the same incubation times.

Regarding cell enumeration, one milliliter of MW or 1 cm^2^ of wood were taken from the batches inoculated with planktonic (pre-adapted and non-pre-adapted cells) and biofilm cells, respectively. The wood supports were scraped with a sterile scalpel in order to remove the adherent cells in form of biofilm. In all cases, the cell samples were diluted in physiological solution (9 g/L NaCl) and the cells were enumerated in MRS agar after incubation for 24 h at 28 °C under anaerobic conditions using an anaerobic system (Oxoid). As far as L-malic acid conversion is concerned, the concentration of L-malic acid and L-lactic acid was evaluated using enzymatic kits (R-Biopharm, Darmstadt, Germany).

### 2.5. Statistical Analysis

Data were analyzed using the RStudio (v3.5.0) environment [41]. Regarding the screening experiments of biofilm assay (see Section 2.2.1), three independent tests were performed and the mean value was used for data elaboration. The OD values, obtained from the CV assay, were first standardized using the R base function *scale()*, and were subsequently analyzed by statistical analysis based on a multivariate approach. In particular, for the data standardization, the OD values were reported in an electronic spreadsheet and organized in term of a numeric matrix with 68 rows and 5 columns. The rows represent the observations (*L. plantarum* strains) and the columns represent the experimental conditions (control, pH 3.5, pH 3.2, Et12, Et14). For each column, mean and standard deviation were calculated. The average value was subtracted from each value and then divided by the value of the standard deviation corresponding to the same column. Standardized data were processed with the aim to investigate the relationship between biofilm production (response variable) and the source of strains isolation, type, and level of stress. For this purpose, the R package ComplexHeatmap (v2.4.2) [42] was used to process the heat-map and the package FactoMineR (v2.1) [43] was used for the principal component analysis (PCA).

The package ggplot2 [44] was used for graphical representation of data. The analysis of variance (ANOVA), coupled with the TukeyHSD post-hoc test, was performed to elaborate the data of the other experiments. The significance level for the statistical tests was set to an α of 0.05.

## 3. Results and Discussion

### 3.1. Effect of Environmental Conditions on Biofilm Formation

**Figure 1:**
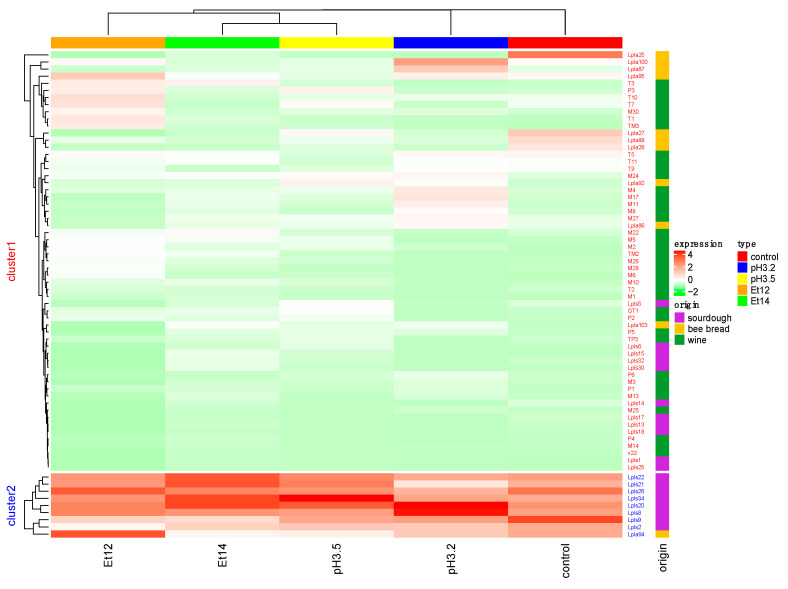
Heat-map related to the production of biofilm (expression) determined on 68 *L. plantarum* strains from different matrices (origin). The strains were grown for 24 h at 28 °C under different stress conditions (type): in Man Rogosa Sharpe (MRS) broth (control), in MRS broth at pH 3.5 (pH 3.5), in MRS broth at pH 3.2 (pH 3.2), and in MRS broth containing ethanol at 12% (Et12) or ethanol at 14% (Et14).

The ability to produce biofilm in hard environmental conditions by *L. plantarum* strains isolated from different matrices was investigated. The effect of both stress conditions and isolation sources on *L. plantarum* metabolic expression and biofilm formation is still unclear. In fact, regarding the variable “stress conditions”, Aoudia et al. [32] evaluated the ability of *L. plantarum* to form biofilm, reporting that acid stress led to a delay in biofilm formation. Instead, other authors reported that the production of biofilm may be induced by environmental conditions [45]. In relation to the variable “origin”, several authors [46,47,48] highlighted that foods or environments characterized by harsh conditions (antimicrobial substances and low pH) harbor a higher number of resistant and virulent microorganisms compared with other non-stressful environments. In our study, the relationship between the ability to form biofilm was evaluated in relation to some environmental stress conditions and different isolation sources by cluster analysis. Specifically, tests performed on 68 strains of *L. plantarum* incubated for 2 h in conventional (control) or in acid (pH 3.5, pH 3.2) or ethanol (Et12, Et14) stress conditions all showed low biofilm formation (data not shown). In contrast, after 24 h of incubation (Figure 1), 59 strains still showed low biofilm formation (cluster 1), while 9 strains (cluster 2) displayed a strong ability to form biofilm. The results in Figure 1 are reported as biofilm expression (calculated as reported in Section 2.5) assuming values from −2 to +4. In particular, negative values correspond to raw values lower than the average values, while positive values indicate raw values higher than the average values.

Moreover, the heat map also highlighted that the biofilm formation did not depend on the type and presence of stress conditions. In fact, the strains grouped in cluster 2 were able to produce biofilm regardless to the presence of stress conditions. This statement is in accordance with other studies that highlighted that some strains of lactic acid bacteria are naturally prone to form biofilm [45,49]. As for the effect of the variable “origin” on the biofilm, some authors evidenced that the biofilm formation is usually affected by various factors such as environmental features, nutrient availability, and the presence of specific matter [50,51,52]. In our study, we found that cluster 2 (Figure 1) enclosed only strains isolated from sourdough and bee bread and no strains isolated from traditional red wines. On the other hand, 11 strains from sourdough, 9 from bee bread, and all the strains isolated from wine as well as the commercial culture *L. plantarum* V22 (Lallemand Inc., Montreal, QC, Canada) were grouped in cluster 1 (low biofilm producers). This fact raised doubts on the influence of the variable “origin” on the ability of strains to form biofilm.

**Figure 2:**
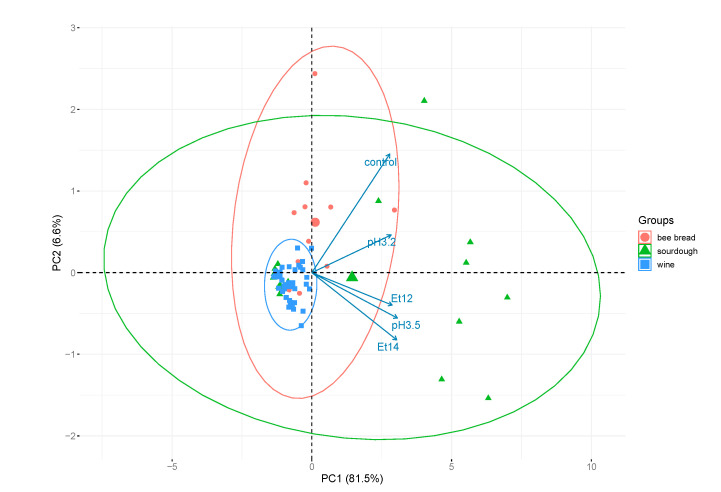
Principal component analysis (PCA) biplot of the first two principal components grouped (groups) by matrix of isolation.

For this reason, principal component analysis (PCA) was applied (Figure 2), showing that biofilm forming activity was not correlated with the isolation source. Specifically, the effect of tested variables on the general behavior of data was explained (88.1%) by two principal components, of which about 82% was attributable to component 1 (PC1).

These data highlight that the ability of *L. plantarum* to form biofilm is a strain-dependent character, unaffected by origin sources or by external factors.

### 3.2. Effect of Incubation Time on the Biofilm Production

**Figure 3:**
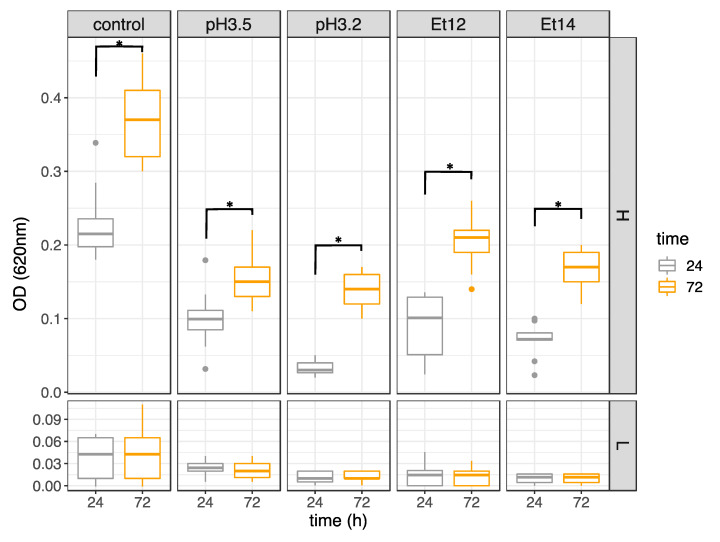
Effect of incubation time (24 h vs. 72 h) on *L. plantarum* biofilm formation. Strains are grouped in high (H) and low (L) biofilm producers. * indicates significant differences (*p* < 0.05). OD, optical density.

In order to avoid an underestimation of biofilm formation for low producer strains and to better elucidate the ability to form biofilm in high producer ones, the effect of a prolonged incubation time up to 72 h in stress conditions was considered on *L. plantarum* biofilm formation. The results, reported in Figure 3, confirmed the division of strains into two groups, named high (group H) and low (group L) biofilm producers, corresponding to those belonging to cluster 2 and cluster 1 in Figure 1, respectively. The low ability to form biofilm was confirmed for 59 strains, including all the strains isolated from wine and the commercial culture V22. Specifically, regardless of the incubation time, strains grouped in L highlighted poor biofilm formation in absence (control) and in presence of acid (pH 3.5, pH 3.2) or ethanol (Et12, Et14) stress conditions. These strains, even if characterized by a viability in planktonic form similar to that evidenced by strains grouped in H, actually showed low CV OD values in all assayed conditions.

Conversely, the biofilm production of strains grouped in H was strongly affected by the incubation time, and in all the conditions, a significant increase (*p* < 0.05) in biofilm production was observed after 72 h compared with 24 h. This last evidence is consistent with findings observed by Ramírez et al. [33], who reported the positive effect of prolonged incubation time on the biofilm maturation.

Data reported in Figure 3, panel H, seem to be inconsistent with those given in Figure 1, cluster 2. In fact, the former evidenced that biofilm formation was not dependent on the type and presence of stress conditions, while the latter showed that the quantity of biofilm was significantly (*p* < 0.05) lower in presence of acid (pH 3.5, pH 3.2) or ethanol (Et12, Et14) stress conditions with respect to that observed in the control.

**Figure 4:**
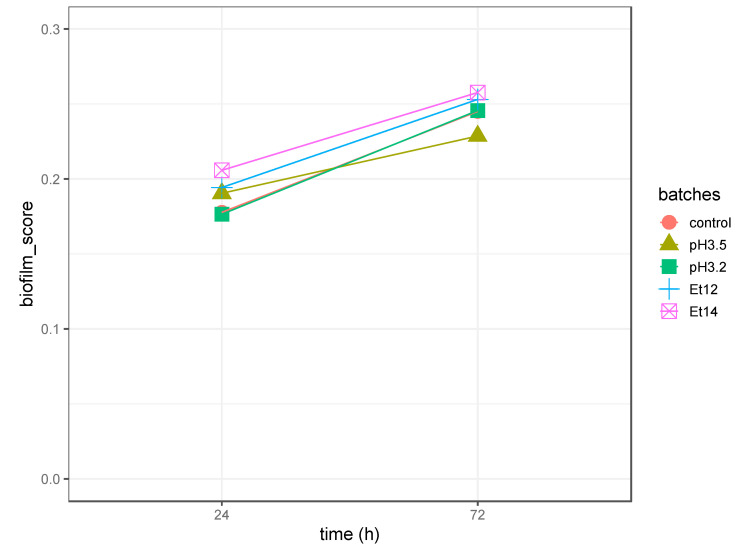
Biofilms produced (biofilm_score) by high producer strains of *L. plantarum* after 24 h and 72 h of incubation in MRS broth in presence of acid stress (pH 3.5 pH 3.2), ethanol stress (Et12, Et14) or without stress (control). The biofilm_score value is the ratio between the median values of crystal violet (CV) OD_620_ (median values of OD_620_ calculated on cells in biofilm form) and OD_620_ (median values of OD_620_ calculated on cells in planktonic form).

This discrepancy was explained considering the growth rate in planktonic form of high biofilm producer *L. plantarum* strains cultivated in the absence (control) and presence of acid (pH 3.5, pH 3.2) or ethanol (Et12, Et14) stress. In fact, the significant differences (*p* < 0.05) in planktonic growth rate were dependent on the cultural conditions (Table S1). When the strains were cultivated in the control batch, a maximum growth rate value of about 0.45 h^−1^ was observed, while the strains cultured in low acid (pH 3.2) or high ethanol (Et14) stress conditions exhibited maximum growth rate values of about 0.08 h^−1^ and 0.12 h^−1^, respectively. The differences in growth rate also affected the final amounts of cells in the diverse cultural conditions. In fact, the lowest OD value (0.18) at 24 h was detected for the strains in acid (pH 3.2) stress conditions and at 72 h OD values of about 0.55 and 0.56 were found for the strains in acid (pH 3.2) and ethanol stress conditions (Et12), respectively. Conversely, the OD detected for the strains in control conditions reached values of about 1.0 at 24 h and 1.3 at 72 h. For this reason, OD values from CV assay were normalised, taking into account the OD of the cells in planktonic forms, obtaining a dimensionless parameter arbitrary labelled as “biofilm_score”. In detail, the biofilm_score was calculated as the ratio between the median values of CV OD_620_ (median values of OD_620_ calculated on cells in biofilm form) and OD_620_ (median values of OD_620_ calculated on cells in planktonic form). As shown in Figure 4, after 24 h of incubation, the biofilm_score assumed values between 0.16 and 0.21 and no significant difference was detected among the different cultivation conditions (control, ethanol, and acid stress conditions). Slightly higher values in the biofilm_score were detected after 72 h of incubation. Therefore, the presence or the absence of stress conditions and the type of stressors did not influence the biofilm_score and likely the biofilm formation in the assayed *L. plantarum* strains. Slight differences in biofilm_score derived only from an increased incubation time, and this fact can be explained by an increase in extracellular substances, as well documented by others authors [53,54]. In fact, it should be considered that the biofilm matrix is a complex structure, which, in addition to cells, encloses several extracellular compounds such as proteins, exopolysaccharides, and other extracellular substances. These substances can be bound by CV [33], explaining higher OD_620_ values after 72 h with respect to those detected after 24 h of incubation.

### 3.3. Culturable Cells in the Biofilm

**Figure 5:**
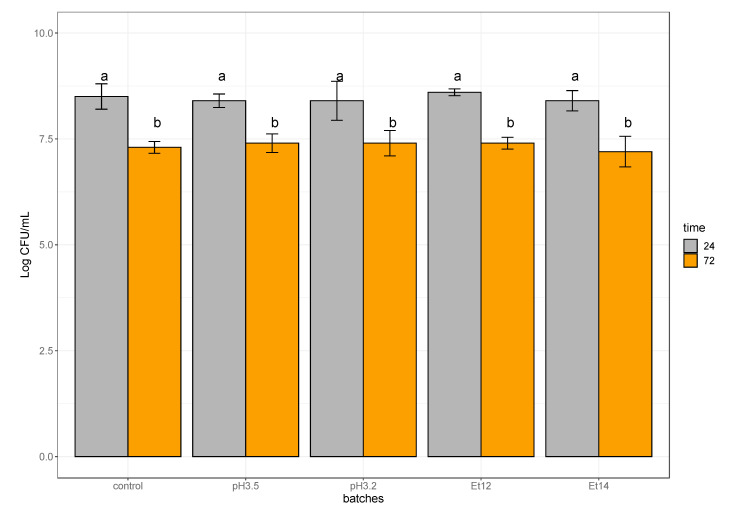
Mean values with standard deviation of *L. plantarum* strains belonging to high biofilm producer group. Cells were enumerated in the biofilms of 24 and 72 h, formed in MRS broth in presence of acid stress (pH 3.5, pH 3.2), ethanol stress (Et12, Et14), or without stress (control). Different letters indicate significant differences (*p* < 0.05).

Culturable cells in biofilm formed by *L. plantarum* high producer strains were evaluated at early (24 h) and at late (72 h) stage. Regardless of the stress conditions (*p* > 0.05), in the biofilms at early stage, an amount of approximately 8.5 Log CFU/mL was detected (Figure 5). Likewise, also in biofilms at late stage, no significant differences (*p* > 0.05) between control and stress conditions were detected, but culturable cells enumerated in biofilms were significantly lower (*p* < 0.05) than those recorded in the biofilm at early stage. In fact, a decrease of about 1 Log CFU/mL was recognised after 72 h of incubation. The culturable cell decay could be the result of different factors, such as their entering in a viable but non-culturable state (VBNC), the behavior of cells in biofilm, and their specific aggregations. In fact, the formation of bacterial cell aggregates could give rise to a single colony on plate, resulting in an underestimation of culturable cells [18,55]. Furthermore, cells in a mature biofilm can also enter in a VBNC state, resulting in being unable to grow in routine bacteriological culturing media, while maintaining their metabolic activity [56]. This cell condition is widely documented for several *Lactobacillus* species, including *L. plantarum*, in certain extreme environments [54,57,58,59].

Independently from the cell state (VBNC or culturable cells), the effectiveness of biofilm on malolactic fermentation was clarified in the present study using a specific model wine.

### 3.4. Impact of Biofilms and Planktonic Cells of L. plantarum Lpls22 on the Degradation of L-malic Acid in Model Wine

**Figure 6:**
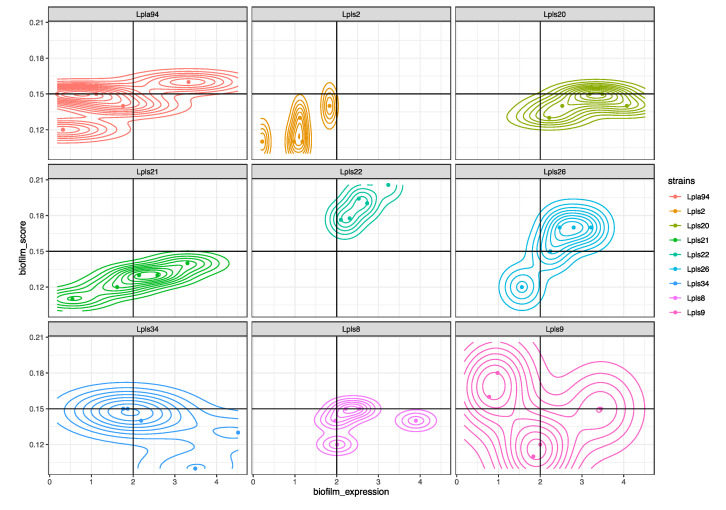
Density plot of biofilm_expression and biofilm_score indices of high biofilm *L. plantarum* producer strains.

On the basis of previous experiments, the strain *L. plantarum* Lpls22 was selected as the highest performing producer strain. More in detail, all high biofilm producer strains were compared for their biofilm expression values and specific biofilm_score values. As highlighted in Figure 6, all the specific biofilm_score and biofilm_expression plot points of *L. plantarum* Lpls22 were in the comfortable zone.

Model wine at 12% ethanol and at two different pH values (3.2 or 3.5) was used to assess the survival of culturable cells in biofilm and in planktonic forms. For this purpose, each batch of model wine was inoculated with biofilm attached to wood supports or with cells in planktonic form.

**Figure 7:**
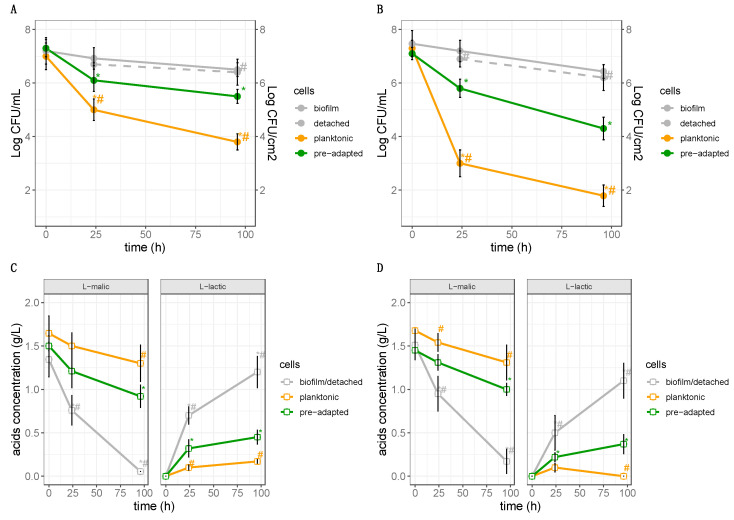
Survival of planktonic cells, biofilm cells, detached cells from biofilm, and pre-adapted cells of *L. plantarum* Lpls22 inoculated at high concentration in MW at (**A**) pH 3.5 and (**B**) pH 3.2. Trend of L-malic and L-lactic acid in MW at (**C**) pH 3.5 and (**D**) pH 3.2 inoculated with biofilm cells, planktonic cells, or pre-adapted cells of *L. plantarum* Lpls22. Data are reported as mean values with standard deviation of three biological replicates. * indicates significant (*p* < 0.05) differences compared with 0 h. # indicates significant (*p* < 0.05) differences compared with pre-adapted cells at the same time.

More in detail, 10 wood supports with attached biofilm (≃7 Log cells/cm^2^) were used as inoculum obtaining ≃10^9^ cells in 240 mL of MW, corresponding to about 7 Log CFU/mL. Consequently, the cells in planktonic form were inoculated in MW in order to obtain final concentrations of about 7 Log CFU/mL. Moreover, control batches were inoculated with *L. plantarum* Lpls22 in planktonic form, pre-adapted as reported by Succi et al. [29], in order to obtain the same final concentrations (about 7 Log CFU/mL). The inoculum concentrations appeared closely consistent with literature. In fact, to date, several authors consider cell levels between 10^7^ and 10^8^ CFU/mL of model wine or wine as the optimal inoculum concentration [29,60,61]. The survival was monitored for 96 h at 20 °C. The results, reported in Figure 7A,B show the survival of planktonic and biofilm cells as well as cells detached from biofilm. The data highlighted that culturable cells in planktonic form were characterized by a strong cell decay (*p* < 0.05) already after 24 h of incubation, as evidenced by a decrease of about 2 Log CFU/mL and 4 Log CFU/mL at pH 3.5 and 3.2, respectively. At the end of the incubation time (96 h), a further decay characterized the planktonic cells, as shown by a decrease of about 3 Log CFU/mL and 5 Log CFU/mL at pH 3.5 and 3.2, respectively.

The reduction registered for cells in planktonic form could adversely affect the enzymatic activities such as the conversion of L-malic acid to L-lactic acid. In fact, as reported in the literature, MLF starts when the LAB population reaches 6 Log CFU/mL [62]. In agreement with other studies [25,29], the results obtained showed that the survival of *L. plantarum* is strongly affected by a high ethanol level (12%) combined with a low pH (both pH 3.2 and pH 3.5). Conversely, biofilm life-style is widely recognized as a suitable tool to protect bacteria from harsh environmental conditions [31,63]. In agreement with these findings, in our work, the levels of culturable cells attached to biofilm were not significantly affected (*p* > 0.05) in MW at pH 3.5 and pH 3.2, highlighting that cells of *L. plantarum* in biofilm form were much more resistant than planktonic ones. Moreover, cells detached from biofilm were characterized by count levels similar to those found in biofilm attached cells. This finding could be because of the similar molecular response in both biofilm cells and biofilm detached cells. In fact, as reported by Guilhen et al. [64], cells dispersed from biofilms have a high stress response because they are transcriptionally closer to their parent cells in biofilm form than to cells in planktonic form. Moreover, the survival of cells in biofilm life-style, both attached or detached, was significantly higher than those evidenced by the pre-adapted cells of *L. plantarum* Lpls22. Our study also considered the effect of *L. plantarum* biofilm life-style on L-malic acid decarboxylation. So far, biofilms produced by *L. plantarum* strains have been widely investigated for their negative impact on foods [33,35,54]. In other studies, extracellular substances from *L. plantarum* were characterized and specific exopolysaccharides were suggested as food adjunct for their functional or technological roles [65,66]. However, the relationship between biofilm formation by *L. plantarum* and the enhancement of MLF is poorly investigated. Positive effects of biofilm life-style on MLF were reported for a *Oenococcus oeni* [38]. In our study, the behaviour of L-malic acid and L-lactic acid highlighted for the first time the effectiveness of *L. plantarum* cells in biofilm form on wine biological decarboxylation. Instead, no L-malic acid consumption was detected in MW containing planktonic cells. Similarly, a significant (*p* < 0.05) increase in levels of L-lactic acid was only found in MW with biofilm. Therefore, the survival of attached or detached *L. plantarum* cells in biofilm life-style strongly affects the malic decarboxylation. As reported in the literature, *L. plantarum* in acid stress conditions and in the presence of malic acid could use malate decarboxylase, also known as malolactic enzyme, for the direct conversion of malic acid to lactic acid [67]. In addition, the results showed that *L. plantarum* in biofilm life-style allows to reach performances higher than those obtained by acclimated *L. plantarum* strains.

In conclusion, it is possible to assert that *L. plantarum* in biofilm form could be considered as a suitable biotechnological strategy to optimize the malolactic fermentation. To date, the preadaptation to stress conditions was considered as an important method to optimize the survival of oenological strains in wine environments [68,69]. Considering the results obtained in this study, in our opinion, the knowledge regarding the cell efficacy, in terms of survival and MLF, of useful *L. plantarum* strains was strongly enriched. This achievement certainly represents the main result. Furthermore, other important and innovative aspects for the screening of malolactic *L. plantarum* strains were introduced. The biofilm formation, as evidenced by the assayed strains, is a strictly strain-dependent character. The results clearly highlighted that neither the stress environmental conditions nor the origin influence the biofilm formation. This evidence has two meanings in the screening of malolactic strains. On the one hand, a high number of *L. plantarum* strains from different sources, not only from certain environments, must be considered in the selection and primary screening, as the strain origin does not influence the biofilm formation. On the other hand, considering that biofilm formation is unaffected by stress conditions, the screening process could be performed using faster approaches without testing the strain performance in several environmental conditions. In addition, the use of *L. plantarum* malolactic strains in biofilm life-style represents an alternative strategy to some time-consuming approaches, such as culture preadaptation. On the basis of these considerations, the scale up of the use of biofilm attached to wooden support in winemaking pilot or industrial scale could be further developed also considering their contribution to wine aroma.

## Supplementary Materials

The following are available online at https://www.mdpi.com/2304-8158/9/6/797/s1, Table S1: Kinetic parameters obtained from the cultivation of high biofilm producer strains of *L. plantarum* in planktonic form.
