# Supplementary material for: Effect of Biofilm Formation by Lactobacillus plantarum on the Malolactic Fermentation in Model Wine
Source: Foods. 2020 Jun 17;9(6):797. doi: 10.3390/foods9060797 (PMC7353508; doi:10.3390/foods9060797)
Supplement: Supplementary file 1 [file foods-09-00797-s001.pdf]

TableS1. Kinetic parameters obtained from the cultivation of high biofilm producer strains of *L. plantarum* in planktonic form.

Batches	μ_{max} (h^{-1})	OD_{24h}	OD_{72h}
Control	0.43 ± 0.03	1.05 ± 0.05	1.32 ± 0.13
pH3.5	0.32 ± 0.01	0.58 ± 0.11	0.75 ± 0.03
pH3.2	0.08 ± 0.02	0.18 ± 0.03	0.55 ± 0.03
Et12	0.27 ± 0.02	0.41 ± 0.05	0.80 ± 0.07
Et14	0.12 ± 0.04	0.30 ± 0.03	0.56 ± 0.06

Mean values of 9 strains ± SD
